# Bioinformatics analysis identifies key secretory protein-encoding differentially expressed genes in adipose tissue of metabolic syndrome

**DOI:** 10.1080/21623945.2024.2446243

**Published:** 2025-01-16

**Authors:** Jiandong Zhou, Yunshan Guo, Xuan Liu, Weijie Yuan

**Affiliations:** Department of Nephrology, Shanghai General Hospital of Nanjing Medical University, Shanghai, China

**Keywords:** Secretory proteins, epigenetics, adipose tissue, metabolic syndrome, bioinformatics

## Abstract

The objective of this study was to identify key secretory protein-encoding differentially expressed genes (SP-DEGs) in adipose tissue in female metabolic syndrome, thus detecting potential targets in treatment. We examined gene expression profiles in 8 women with metabolic syndrome and 7 healthy, normal body weight women. A total of 143 SP-DEGs were screened, including 83 upregulated genes and 60 downregulated genes. GO analyses of these SP-DEGs included proteolysis, angiogenesis, positive regulation of endothelial cell proliferation, immune response, protein processing, positive regulation of neuroblast proliferation, cell adhesion and ER to Golgi vesicle-mediated transport. KEGG pathway analysis of the SP-DEGs were involved in the TGF-beta signalling pathway, cytokine‒cytokine receptor interactions, the hippo signalling pathway, Malaria. Two modules were identified from the PPI network, namely, Module 1 (DNMT1, KDM1A, NCoR1, and E2F1) and Module 2 (IL-7 R, IL-12A, and CSF3). The gene DNMT1 was shared between the network modules and the WGCNA brown module. According to the single-gene GSEA results, DNMT1 was significantly positively correlated with histidine metabolism and phenylalanine metabolism. This study identified 7 key SP-DEGs in adipose tissue. DNMT1 was selected as the central gene in the development of metabolic syndrome and might be a potential therapeutic target.

## 1. Introduction

Metabolic syndrome is a complex metabolic disorder syndrome related to insulin resistance, including obesity, hypertriglyceridaemia, low high-density lipoprotein cholesterol, hypertension, diabetes, and impaired blood glucose regulation [1]. In fact, it is believed that weight gain, especially central or abdominal obesity, is at the core of the pathogenesis of metabolic syndrome [2]. According to epidemiological statistics, approximately one-fourth of the world’s population will suffer from metabolic syndrome [3]. Metabolic syndrome is associated with an increased incidence of diabetes and a greater risk of cardiovascular events such as heart disease and stroke, making it a major public health issue [4]. Therefore, effectively preventing and treating metabolic syndrome is urgent.

Adipose tissue plays an important role in the occurrence and development of metabolic syndrome. Adipose tissue consists of mature adipocytes, preadipocytes, adipose tissue macrophages, other immune cells, neurons, progenitor cells, fibroblasts, endothelial cells, epithelial cells and stromal vascular cells. Central obesity can lead to adipocyte hypertrophy and hyperplasia, macrophage infiltration, and endothelial cell activation. The expansion of adipose tissue increases the production of metabolic products, including adipocyte-derived peptide hormones, inflammatory mediators, signalling lipids, and miRNAs packaged in exosomes, which are broadly associated with atherosclerosis, endothelial dysfunction, hypertension, and dyslipidemia. Obesity not only increases the number of macrophages in adipose tissue but also triggers the polarization of M1 macrophages, resulting in the production of more proinflammatory cytokines and leading to insulin resistance [5,6]. Adipocyte hypertrophy and hyperplasia can also cause local hypoxia, leading to decreased adiponectin production and increased expression of proinflammatory cytokines [7]. In this situation, adipokines and cytokines play important roles in chronic inflammation, macrophage accumulation, and hypoxia, leading to the occurrence of metabolic syndrome.

Adipose tissue is an important endocrine organ that causes metabolic syndrome [8]. Adipose tissue releases many protein hormones and signalling factors, promoting communication between adipose tissue and other organs [9,10]. It plays a role in regulating metabolic processes through paracrine and endocrine signalling in health and disease [11]. Previous studies have indicated that fat tissue is associated with insulin resistance, lipoprotein metabolism, and elevated blood pressure [12]. Several factors secreted by adipocytes and macrophages in adipose tissue promote the development of metabolic syndrome [6,13]. The chronic inflammatory state associated with central obesity is considered the main factor in the development of metabolic syndrome and its related pathophysiological consequences [14]. Adipocytes, as endocrine organs, produce lipid metabolic products, hormones, and chemotactic factors to coordinate systemic glucose and lipid metabolism [15]. Released adipokines include hormones (such as leptin and adiponectin), peptides (such as angiotensinogen, apelin, resistin, and plasminogen activator inhibitor (PAI)-1), and inflammatory cytokines (such as interleukin (IL)-6, tumour necrosis factor-α (TNFα), adiponectin, reticulum protein and chemerin), all of which play important roles in the pathophysiology of insulin resistance and metabolic syndrome [16]. In released hormones, the level of leptin is directly proportional to obesity and body fat levels. When the body’s energy reserves are sufficient, leptin suppresses food intake and stimulates energy expenditure while controlling glucose homoeostasis and insulin sensitivity. However, high levels of leptin fail to correct the metabolic imbalance in obesity, leading to the concept of ‘leptin resistance’, where tissues have reduced sensitivity to leptin, leading to disorders of glucose and lipid metabolism and insulin resistance [17]. Adipose tissue can also produce the peptide angiotensin II (Ang II), which increases the production of reactive oxygen species (ROS). ROS have multiple effects, including endothelial damage, NF-kB expression, and the expression of lipid receptor-1 (LOX-1) in VSMCs and endothelial cells [18]. RAS, ROS, and LOX-1 form a vicious cycle, inducing endothelial dysfunction, inflammation, and fibroblast proliferation, leading to the progression of dyslipidemia, T2DM, hypertension, and cardiovascular disease [19]. Therefore, adipose tissue plays an important role in the occurrence and development of metabolic syndrome. However, the pathophysiological mechanisms of metabolic syndrome have not been thoroughly studied, so exploring the role of proteins secreted from adipose tissue is highly important for elucidating the molecular characteristics and mechanisms of the occurrence and development of metabolic syndrome.

Microarray technology is used to detect gene expression, and bioinformatics analysis tools are used to explore potential pathogenesis and pathogenic mechanisms at the genomic level. This is an effective method for large-scale research on gene expression, and increasing evidence shows that genetic factors play a key role in the progression of metabolic syndrome. Previous studies involving high-throughput transcriptome data and comprehensive bioinformatics identified nine key genes associated with metabolic syndrome in peripheral blood, including SPTAN1, KCTD7, PSMD1, FZD1, KLHL9, PTTG1, TSPAN14, P2RY2, and CXCR5 [20]. The genomic DNA of blood samples from metabolic syndrome using the MEDISCOPE chip containing 758,000 SNPs identified that MUT, AACS, SLC6A15 and PRKCA genes are associated with insulin resistance of metabolic syndrome and involve in branched chain amino acid metabolism or regulation [21]. A study of the miRNA‒mRNA regulatory network and key genes related to metabolic syndrome in peripheral blood revealed that the transcription factors SP1, SP4, and EGR1 mediate the upregulation of DPYSL4 expression induced by miR-34c-5. This pathway may be a potential mechanism for the occurrence and development of metabolic syndrome [22]. A study with a large sample size revealed that five blood metabolites, namely, LysoPC(14:0), LysoPC(15:0), propionyl carnitine, phenylalanine, and docosapentaenoic acid, can serve as biomarkers for metabolic syndrome, and three SNPs (rs11635491, rs1952458, and rs7067822) are associated with the levels of LysoPC(15:0) [23]. Another study suggested that the altered expression of clock genes in obese visceral adipose tissue play an important role in the development of metabolic syndrome [24]. Owing to the difficulties in studying the secretion of adipose tissue [8], few studies have explored the genes related to metabolic syndrome from the perspective of adipose tissue, providing a thorough understanding of the key genes involved in the pathogenesis of metabolic syndrome.

## 2. Results

### 2.1. Data preprocessing and DEGs analysis

**Figure 1.**
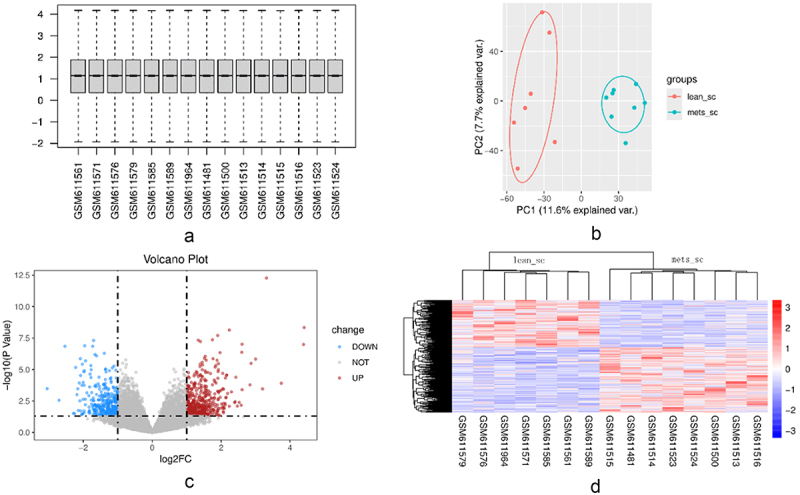
Results after processing the chip expression data. Figure 1a: after normalization, the median expression of each chip was at the same level, eliminating errors from other interference experiments. Figure 1b: Principal component analysis (PCA) used to cluster the 15 samples. Figure 1c: volcano plot of the differentially expressed genes (DEGs); the red dots indicate the genes whose expression is upregulated, and the blue dots indicate genes whose expression is downregulated in the adipose tissue of patients with metabolic syndrome. Figure 1d: Heatmap of the distribution of the DEGs in the 15 samples. Red indicates the upregulated DEGs, and blue indicates the downregulated DEGs. Each column represents a sample, and each row represents a DEG.

We conducted bioinformatics analysis using the GSE24883 dataset and selected 15 subcutaneous adipose tissue samples from 7 healthy, normal weight females and 8 females with metabolic syndrome. The data was corrected using the ‘normalizeBetweenArrays’ function in the ‘limma’ package to obtain the boxplot (Figure 1a). The results of principal component analysis (PCA) showed that the metabolic syndrome samples were completely separated from the normal weight controls (Figure 1b). Eight hundred and twenty-two differentially expressed genes (DEGs) were identified, including 478 upregulated genes and 344 downregulated genes. The analysis results are shown in a volcano plot (Figure 1c) and a heatmap (Figure 1d).

### 2.2. Identification of the SP-DEGs

**Figure 2.**
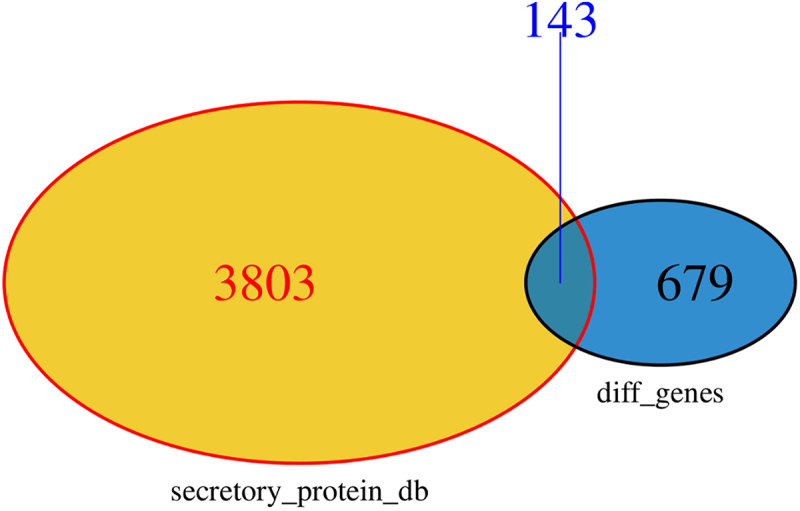
Venn diagram of the SP-DEGs and human secretory protein-encoding gene database.

**Figure 3.**
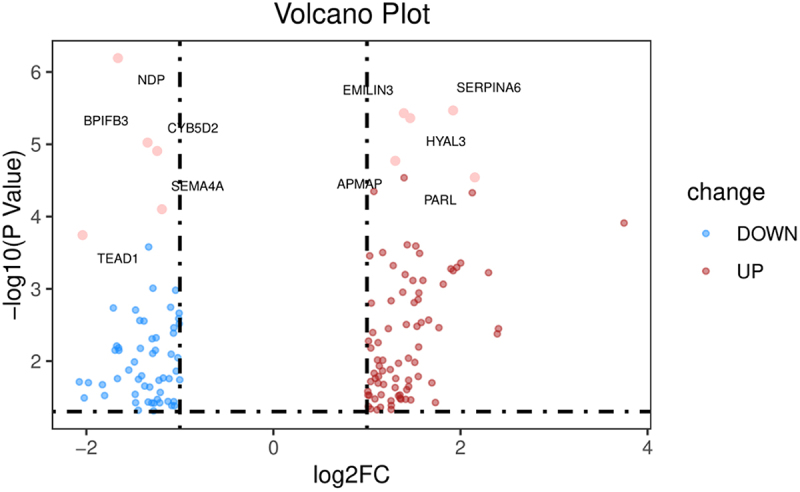
Volcano plot of the 143 SP-DEGs; the red dots represent the upregulated genes, and the blue dots represent the downregulated genes in the adipose tissue of patients with metabolic syndrome.

The secretory protein dataset was downloaded from the Human Protein Atlas database (https://www.proteinatlas.org/). A Venn diagram (Figure 2) was used to obtain the intersection of 822 DEGs and the protein_class_SPOCTOPUS dataset, and 143 secretory proteins were obtained, including 83 upregulated and 60 downregulated genes. See Supplement Table 1. A volcano plot (Figure 3) was used to visualize the 143 differentially expressed protein-encoding genes.

**Table 1.** GO BP enrichment and KEGG pathway analysis of module 1 and module 2 (*p* < 0.05).

	Term	Description	Count	P value
Module 1	GO:0000122	negative regulation of transcription from RNA polymerase II promoter	4	1.44E–04
	GO:0045892	negative regulation of transcription, DNA-templated	3	0.00259
	GO:0043392	negative regulation of DNA binding	2	0.00311
	GO:0006351	transcription, DNA-templated	2	0.01565
	hsa01522	Endocrine resistance	2	0.03356
Module 2	GO:0006955	immune response	3	6.62E–04
	GO:0050830	defense response to Gram-positive bacterium	2	0.01325
	GO:0019221	cytokine-mediated signalling pathway	2	0.01521
	hsa04630	JAK-STAT signalling pathway	3	3.65E–04
	hsa04060	Cytokine-cytokine receptor interaction	3	0.00117
	hsa05144	Malaria	2	0.01151
	hsa04640	Hematopoietic cell lineage	2	0.02273

### 2.3. Functional enrichment and pathway analysis of the SP-DEGs

**Figure 4.**
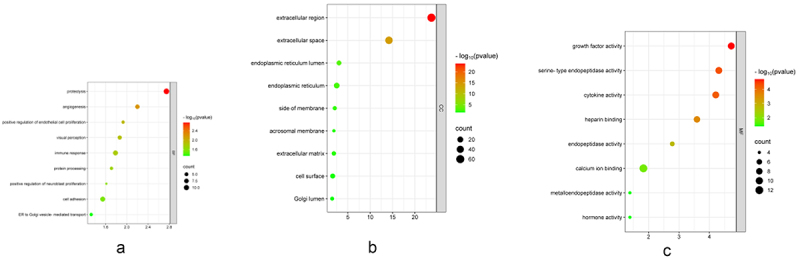
GO functional enrichment analysis of the SP-DEGs. According to the ascending order of the *p* values, the nodes are displayed in a gradient from red to green based on their colour, whereas the node size is displayed on the basis of the gene counts (*p* values < 0.05 and counts ≥3). Figure 4a: enrichment results for biological processes. Figure 4b: enrichment results for cellular components. Figure 4c: enrichment results for molecular functions.

**Figure 5.**
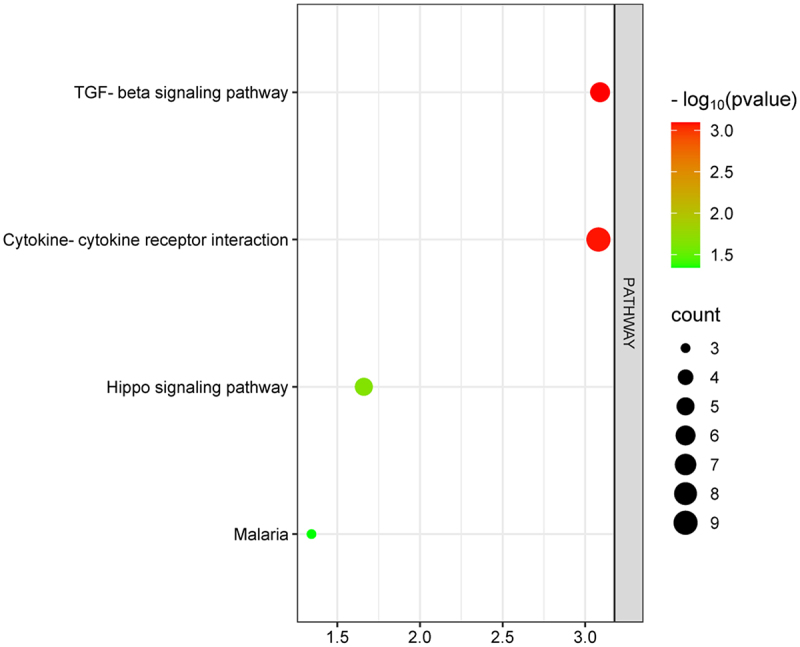
KEGG pathway analysis of the SP-DEGs.

In the GO analyses, the ‘biological process’ terms of these SP-DEGs included proteolysis, angiogenesis, positive regulation of endothelial cell proliferation, immune response, protein processing, positive regulation of neuroblast proliferation, cell adhesion and ER to Golgi vesicle-mediated transport (Figure 4a), and the ‘cellular component’ terms were associated mainly with the extracellular region, extracellular space, endoplasmic reticulum lumen, endoplasmic reticulum, side of membrane, acrosomal membrane, extracellular matrix, cell surface and Golgi lumen (Figure 4b). In terms of the ‘molecular function’ terms, the SP-DEGs were associated primarily with growth factor activity, serine-type endopeptidase activity, cytokine activity, heparin binding, endopeptidase activity, calcium ion binding, and metalloendopeptidase activity and hormone activity (Figure 4c). According to the KEGG pathway analysis, the SP-DEGs were involved in the TGF-beta signalling pathway, cytokine‒cytokine receptor interactions, the hippo signalling pathway, and Malaria (Figure 5).

### 2.4. PPI network construction and module analysis of the SP-DEGs

**Figure 6.**
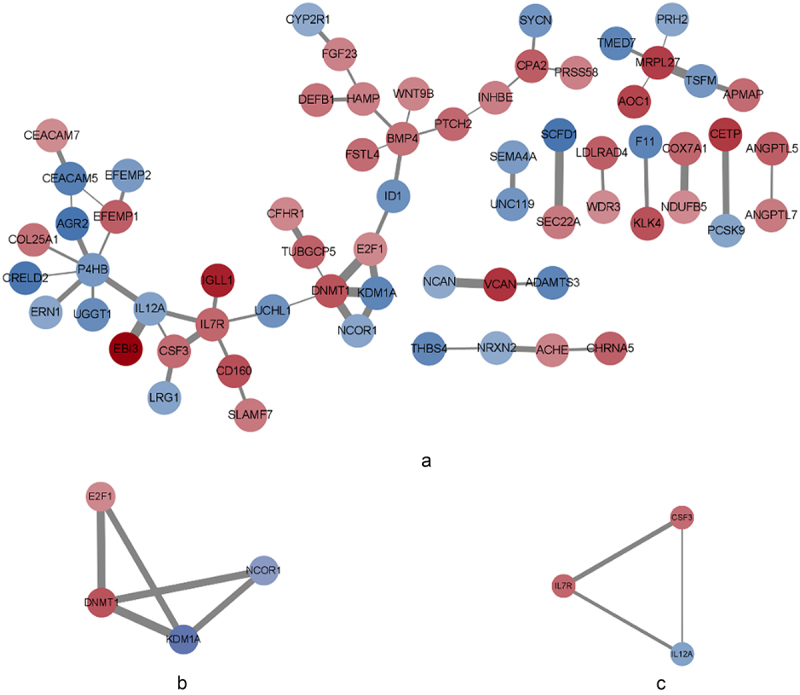
PPI network of the SP-DEGs and modules. Figure 6a: PPI network of the SP-DEGs generated via Cytoscape software; red indicates the upregulated genes, and blue indicates the downregulated genes in metabolic syndrome patients. The lines between the different nodes represent associations between potential protein targets, and the width of the line represents the strength of the interaction. Figure 6b: module 1. Figure 6c: module 2.

In the PPI network, we obtained two modules with high scores, namely, Module 1(DNMT1, KDM1A, NCoR1, and E2F1) and Module 2(IL-7 R, IL-12A, and CSF3). See Figure 6. The GO ‘biological processes’ terms of the Module 1 gene set included negative regulation of transcription from the RNA polymerase II promoter; negative regulation of transcription, DNA-templated; negative regulation of DNA binding; and transcription, DNA-templated. The KEGG pathway analysis showed that the Module 1 gene set was involved in endocrine resistance. The GO ‘biological processes’ terms of the Module 2 gene set included the immune response, the defence response to gram-positive bacteria, and the cytokine-mediated signalling pathway. The KEGG pathway analysis results showed that the Module 2 gene set was mainly involved in the JAK-STAT signalling pathway and cytokine‒cytokine receptor interactions. See Table 1. These results show that the adipose tissue of metabolic syndrome patients participates in metabolism, the immune response, and inflammation.

### 2.5. WGCNA of the gene expression matrix

**Figure 7.**
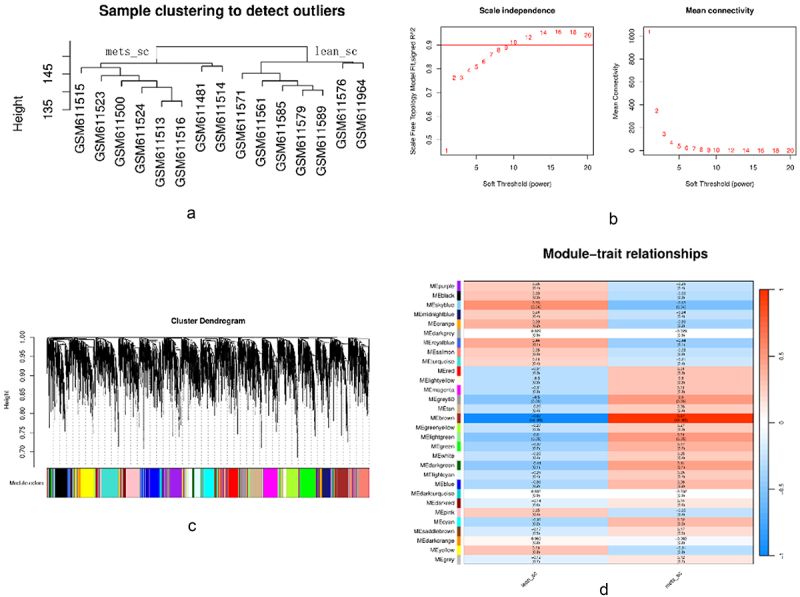
Results of the WGCNA. Figure 7a: sample clustering used to detect outliers. Figure 7b: the left panel shows the scale-free index (y-axis) as a function of the soft threshold power (x-axis). The right panel shows the average connectivity (degree, y-axis) as a function of the soft threshold power (x-axis). The power was set to 10 for further analysis. Figure 7c: identification of the shared gene modules. The branches of the dendrogram are divided into 30 modules, with each module being marked with a unique colour. The relationship between the gene dendrogram and the gene modules was hierarchical. Figure 7d: Heatmap showing the correlation between the characteristic gene set and the phenotypes of each module. The two modules, brown and sky blue, are related to metabolic syndrome, with the brown module being the most relevant to metabolic syndrome.

According to the results of the Pearson correlation and average linkage methods, the sample clustering analysis did not reveal any outliers, as shown in Figure 7a; the correlation between samples was very significant, and all samples were included for further analysis. To achieve scale-free topology (scale-free R^2 > 0.9), a soft threshold of β = 10 was chosen, as shown in Figure 7b. Subsequent WGCNA network construction and average linkage hierarchical clustering revealed 30 gene modules, as shown in Figure 7c; we found that the brown module (|cor| = 0.97, *p* = 4e-09) was significantly positively correlated with metabolic syndrome (Figure 7d). The brown module contains 276 genes. On the basis of the conditions |mM|>0.8 and |GS|>0.2, a total of 55 candidate genes were obtained, as shown in Supplemental Table S2. The DNMT1 gene was shared between the network modules and the WGCNA brown module.

### 2.6. Single-gene GSEA of the core gene

**Figure 8.**
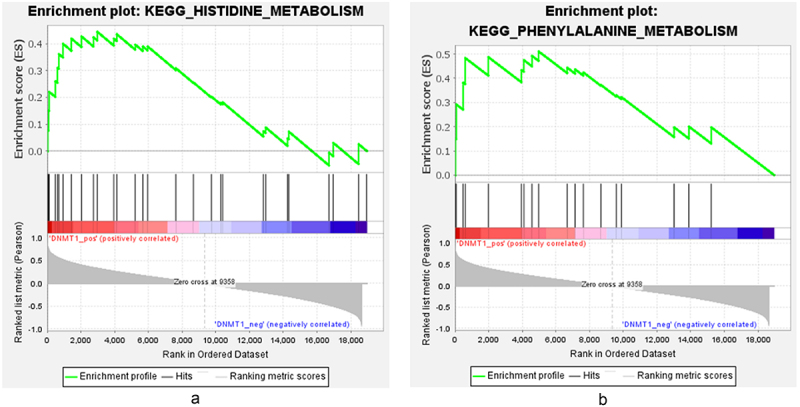
Representative GSEA enrichment score plot of the DNMT1 gene; green represents the enrichment profile. DNMT1 was positively correlated with histidine metabolism (Figure 8a) and phenylalanine metabolism (Figure 8b).

The single-gene GSEA method was used to study the biological functions and related pathways of DNMT1 in the gene expression matrix. DNMT1 was significantly positively correlated with histidine metabolism and phenylalanine metabolism (FDR <0.25, *p* < 0.05, |(NES)| > 1). See Figure 8.

## 3. Discussion

In this study, a series of bioinformatics analyses based on gene expression profiles from GSE24883 were performed. We found that SP-DEGs are mainly involved in metabolism, the immune response, inflammation, angiogenesis and signal transduction. Meanwhile we identified 7 key SP-DEGs, including DNMT1, KDM1A, NCoR1, E2F1, IL-7 R, IL-12A, and CSF3, between adipose tissue samples of patients with metabolic syndrome and healthy controls. We surveyed the biological functions and metabolic pathways of these SP-DEGs via an online website. STRING was used to construct the PPI network of the SP-DEGs, and MCODE was used to identify the two core modules of the PPI network. These SP-DEGs help elucidate the molecular mechanism underlying the pathogenesis of metabolic syndrome, providing a new direction for the treatment of metabolic syndrome. To better understand the role of adipose tissue in the occurrence and development of metabolic syndrome, this study also used WGCNA of gene expression profiles to identify candidate biomarkers for metabolic syndrome. The brown module was significantly positively correlated with metabolic syndrome. DNMT1 was selected as the central gene in the development of metabolic syndrome. Single-gene GSEA revealed that DNMT1 was positively correlated with histidine metabolism and phenylalanine metabolism. Therefore, we speculate that DNMT1 May play an important role in the progression of metabolic syndrome diseases.

Adipocytes can produce a variety of adipokines. Many secretory factors are dysregulated in obesity, type 2 diabetes, and metabolic syndrome, but few comprehensive functional characteristics are known. Here, we focused on the secretory proteins of adipose tissue. This study revealed 83 upregulated secretory proteins involved in proteolysis, the immune response, positive regulation of cell proliferation, the cytokine-mediated signalling pathway, cell adhesion, positive regulation of epidermal cell differentiation, and positive regulation of gene expression in the adipose tissue of patients with metabolic syndrome. This finding is consistent with previous studies on the role of adipose tissue in metabolic syndrome. Two modules were obtained from the PPI network of the 143 secretory proteins, namely, Module 1 (DNMT1, KDM1A, NCoR1, E2F1) and Module 2 (IL-7 R, IL-12A, CSF3). DNMT1 was selected as the central gene in metabolic syndrome.

DNMT1, as the principal DNA methyltransferase in mammalian cells, exhibits the highest abundance among various cell types. It is a large – scale and highly dynamic enzyme possessing multiple regulatory features, which enables it to govern DNA methylation within cells [25]. DNA methylation is an important epigenetic factor that profoundly influences gene activity, including gene expression, gene regulation, and cell differentiation [26]. DNMT1 is a dosage-sensitive gene whose increasing and decreasing levels are both related to phenotypic abnormalities, and maintaining the optimal levels of DNMT1 is crucial for normal development and health [27]. Obesity-induced proinflammatory cytokines promote the expression and enzyme activity of DNMT1. Activated DNMT1 selectively methylates and stimulates the tight chromatin structure in the leptin promoter, hindering the expression of leptin. Inhibiting the activity of DNMT1 with DNMT1 inhibitors can improve obesity-induced glucose intolerance and insulin resistance in a leptin-dependent manner [28]. DNMT1 is associated with increased susceptibility to type 2 diabetes (T2D) in Han Chinese people. In T2D patients, DNMT1 causes high methylation of protein tyrosine phosphatase receptor type delta (PTPRD) DNA and induces insulin signalling silencing [29]. NR4A1 is involved in the insulin signalling pathway, and its expression is significantly reduced in patients with T2D. DNA methylation can silence NR4A1, and DNMT1 inhibition can restore NR4A1 via epigenetic modification. Moreover, the DNMT1 inhibitor aurintricarboxylic acid has a blood sugar-regulating function in T2D [30]. In high-fat and high-sugar-induced adipose tissue, increased levels of DNMT1 play the most important role in the changes in methylation patterns of the Glut4 and leptin genes, which is one of the molecular mechanisms of adipogenesis in C57BL/6J mice [31]. The recruitment and activation of macrophages are considered the core of inflammasome formation in the white adipose tissue of obese patients. In cocultured 3T3-L1 adipocytes, DNMT1 DNA methylation plays a crucial role in regulating macrophage polarization and inflammation. DNMT1-mediated PPARγ1 promotes macrophage DNA methylation, which is significantly enhanced by obesity-related factors such as proinflammatory cytokines, and inhibiting DNA methylation in macrophages suppresses inflammation and improves insulin sensitivity. Mice with bone marrow-specific DNMT1 deficiency (MD1KO) exhibit increased polarization of M2 adipose tissue macrophages (ATMs), inhibition of adipose tissue and systemic inflammation, and protection against obesity-induced insulin resistance. This study helps guide the development of new therapeutic targets for preventing and treating obesity-induced insulin resistance/type 2 diabetes through epigenetic regulation [32]. However, DNMT1 in adipocytes is necessary to maintain the balance of fat and systemic energy, and adipocyte DNMT1 regulates mitochondrial homoeostasis and lipid metabolism by adjusting the expression of DNMT1. The loss of DNMT1 in white adipose tissue causes mitochondrial dysfunction, whereas inhibition of adipocyte DNMT1 promotes excessive lipid accumulation in existing adipocytes and causes expansion of adipose tissue. Targeting all DNMT isoforms with epigenetic drugs for the treatment of obesity and metabolic disorders may lead to unexpected off-target effects, possibly excessive obesity and systemic hyperlipidaemia. Therefore, selectively regulating the action of DNMT1 in adipose tissue may be an effective therapeutic intervention for obesity and metabolic disorders with minimal side effects [33]. This study revealed that DNMT1 is highly expressed in the adipose tissue of patients with metabolic syndrome and that moderately inhibiting the expression of DNMT1 in adipose tissue may be a target for the treatment of metabolic syndrome.

KDM1A, the demethylase of the histone protein complex, inhibits the expression of inflammatory genes in preadipocytes, and diet-induced and genetically obese mouse models show a decrease in KDM1A in adipose tissue, which is associated with increased expression of proinflammatory genes [34]. This study revealed that KDM1A is expressed at low levels in the adipose tissue of patients with metabolic syndrome, indicating a weakened ability to inhibit proinflammatory gene expression. Nuclear receptor corepressor 1/2 (NCoR1/2) and histone deacetylase 3 (HDAC3) are epigenetic regulatory factors that play key roles in gene expression and metabolism and are associated with several metabolic diseases. Deficiencies or dysregulation of NCoR1, NCoR2, and HDAC3 are linked to metabolic diseases such as obesity, type 2 diabetes, and non-alcoholic fatty liver disease. Dysregulation of HDAC3 and the NCoR1/2 complex can lead to the development of metabolic syndrome, including insulin resistance, lipid abnormalities, and obesity [35]. This study revealed that NCOR1 is underexpressed in the adipose tissue of patients with metabolic syndrome and may be involved in the development of metabolic syndrome. E2F1 expression is increased in obese human adipose tissue, and E2F1 participates in regulating the ASK1 (MAP3K5) MAP kinase signalling pathway, autophagy, and TNF superfamily members, linking obesity to the phenotype of inflammation, insulin resistance, and the dysfunction of adipose tissue. E2F1 also regulates miRNA 206 and 210-5p, which may be new targets for alleviating obesity-related blood glucose abnormalities [36]. This study revealed that E2F1 is highly expressed in the adipose tissue of patients with metabolic syndrome and may be involved in insulin resistance. IL-7 R is a regulatory factor in the accumulation and metabolism of lipids in adipose tissue, which triggers inflammation, and has been identified as a key gene in the adipose tissue of obese adults with high blood pressure [37]. IL-7 R is the gene with the highest ranking in the inflammatory pathway and is expressed in both the adipose tissue of obese individuals and atherosclerosis patients [38]. IL-7 R deficiency significantly reduces the body weight and visceral fat weight of mice and improves adipose tissue inflammation and insulin sensitivity [39]. This study revealed high expression of IL7R in the adipose tissue of patients with metabolic syndrome, which may play an important role in promoting the inflammatory response and atherosclerosis. Increased expression of IL-12A and IL-2 in the adipose tissue of obese patients promotes insulin resistance [40]. The expression of IL-12A and CXCL10 in the adipose tissue of obese diabetic patients is also increased, and the expression of IL-12A is significantly positively correlated with the expression of ALOX12, which plays a key role in regulating inflammation [41]. However, research has revealed the complex regulatory effect of TGF-β on the expression of the IL-12A gene, which involves multiple cell signalling pathways, such as the Smad2/3, NF-κB, p38, and JNK1/2 pathways [42]. This study revealed significant enrichment of the TGF-β signalling pathway in adipose tissue, which may lead to low expression of IL-12A in the adipose tissue of patients with metabolic syndrome. This finding is inconsistent with previous research results. In the interstitial blood vessels of the adipose tissue of obese patients, genes such as CSF3 and MCP-1, which attract macrophages, are upregulated during obesity but decrease after weight loss surgery [43]. The promotion of an inflammatory environment with high levels of saturated fatty acids promotes macrophage activation and the expression of related genes, such as CSF3 [44]. This study revealed that CSF3 is highly expressed in the adipose tissue of patients with metabolic syndrome. CSF3 May play an important role in recruiting macrophages and promoting inflammation.

This study has several limitations. First, the sample size of the microarray dataset was not large enough. Second, owing to the limitations of existing data, it is not possible to study the changes in gene expression during disease progression. Further in vitro and in vivo experimental evidence is needed to validate the clinical value of the core gene obtained in this study.

## 4. Methods and materials

The purpose of this study was to screen SP-DEGs in adipose tissue and explain their functions and pathways highly related to the development of metabolic syndrome, providing new ideas for the treatment of metabolic syndrome patients. Bioinformatics was employed to filter and analyse the expression data of gene chips. Subsequently, the differentially expressed genes (DEGs) were analysed, and the secretory protein – encoding differentially expressed genes (SP-DEGs) in abdominal adipose tissue were explored. Thereby, the secretory proteins involved in the pathogenesis of metabolic syndrome were identified. Gene ontology (GO) analysis, Kyoto Encyclopedia of Genes and Genomes (KEGG) pathway analysis, protein‒protein interaction (PPI) network analysis, weighted gene coexpression network analysis (WGCNA), and gene set enrichment analysis (GSEA) of key genes were used to select and annotate candidate genes. R 4.3.2 was used for the data analysis. The pixel of the pictures were adjusted by using Adobe Photoshop CS5.1.

### 4.1. Data download and DEGs identification

The gene expression dataset GSE24883 was downloaded from the GEO database via the following link (https://www.ncbi.nlm.nih.gov/geo/)). GSE24883 uses the Agilent -014,850 Whole Human Genome Microarray 4 × 44K G4112F (feature number version). Fifteen subcutaneous adipose tissue samples from 7 healthy, normal body weight women and 8 women with metabolic syndrome were selected for the dataset. Probe sets without corresponding gene symbols and duplicate gene symbols were removed. When multiple probe sets are mapped to the same gene, the average expression value is retained. Data outliers were detected to ensure the accuracy of the analysis. The gene expression data were standardized before further analysis. Differential gene expression was identified via the R package ‘Limma’(version 3.58.1). Principal component analysis (PCA) was used to visualize the spatial distribution of the samples and evaluate their enrichment level. DEGs were identified on the basis of *p* < 0.05 and log2|Fold change| >1.0. The results were plotted using volcano plots and heatmaps.

### 4.2. Identification of the SP-DEGs

The Human Protein Atlas (https://www.proteinatlas.org/) integrates various omics techniques, including antibody-based imaging, mass spectrometry-based proteomics, transcriptomics, and systems biology, to map human proteins in cells, tissues, and organs. All the data are openly accessible. We downloaded secreted protein-encoding genes (protein_class_SPOCTOPUS) from the Human Protein Atlas database. The protein_class_SPOCTOPUS dataset has a total of 3,946 genes encoding secretory proteins. We obtained the intersection of the DEGs and secreted protein-encoding genes via the Venn diagram tool.

### 4.3. Functional enrichment and pathway analysis of the SP-DEGs

GO is an international standardized gene function classification system that provides a standardized way to annotate gene functions. GO analysis includes three terms: biological process (BP), molecular function (MF), and cellular component (CC). The KEGG database is a biological database that integrates the genome, enzyme pathways, and biochemistry, links genomic information with gene function, displays the connection between genomic information and function, and provides a more intuitive description of metabolic pathways. GO analysis and KEGG pathway of the SP-DEGs were conducted by the Database for Annotation, Visualization and Integrated Discovery(DAVID, https://david.ncifcrf.gov/). GO analysis was used to explore the BP, CC, and MF of SP-DEGs. KEGG pathway analysis was conducted to explore the signalling pathways related to SP-DEGs. *p* values < 0.05 and counts ≥ 3 were considered significantly enriched. The results are visualized on the Microbiology website (https://bioinformatics.com.cn/?keywords=pathway).

### 4.4. PPI analysis of the SP-DEGs

The Search Tool for the Retrieval of Interacting Genes/Proteins (STRING) database (http://string-db.org/) is a repository that provides protein – protein interaction (PPI) data. Protein names corresponding to the selected differentially expressed genes can be input into STRING to obtain an interaction network of encoded proteins in the STRING database and calculate the connectivity of each differentially expressed protein. The SP-DEGs in this study with a score of > 0.4 were input into the PPI network, and a protein‒protein interaction network for evaluating the potential protein interactions of the SP-DEGs was obtained. Visualization was conducted via Cytoscape (version 3.9.0, https://cytoscape.org/). The MCODE plugin was used to cluster these important protein molecules with the default parameters as follows: node score cut-off = 0.2, K-core = 2, and max depth = 100. GO BP and KEGG functional enrichment analyses were performed on the gene sets in module 1 and module 2.

### 4.5. Weighted gene coexpression network analysis (WGCNA) of the gene expression matrix

WGCNA of the gene expression matrix was performed via the R package ‘WGCNA (version 1.6.9)’. We performed sample clustering and selected the top 25% of genes with the most significant differences. Subsequently, the value of the soft threshold parameter was determined, and co expressed gene modules were identified. Moreover, correlations between modules and clinical phenotypes were analysed to determine the modules most relevant to metabolic syndrome. The criteria for module selection were |r| > 0.5 and *p* < 0.05. Finally, we took the intersection of gene sets in the MCODE module and the module most relevant to metabolic syndrome in the WGCNA to obtain core genes.

### 4.6. Gene set enrichment analysis (GSEA) of the core gene

GSEA can be used to determine if a predefined gene set shows statistically significant differences in the gene expression matrix between two different phenotypes. In this study, GSEA software (version 3.0, http://www.gsea-msigdb.org/gsea/index.jsp) was used, while the KEGG pathway gene set (c2.cp.kegg.v7.5.1.symbols) was used as the enrichment background and the core gene was used as the phenotype to calculate Pearson correlation coefficients. The correlations between the core gene and other genes were sorted in descending order of scanning sequences, and the gene sets mentioned above were used as the background genes for the enrichment analysis. The criteria was set as follows: FDR < 0.25, *p* < 0.05, and | (NES)| > 1.

### 4.7. Ethics approval and informed consent

GEO is a public database that encompasses datasets of many studies. The studies in the database has obtained ethical approval and informed consent. Our study is based on the open source data, so there are no ethical issues or other conflicts of interest. The ethics Committee of Wujin People’s Hospital has granted exemption.

## 5. Conclusion

In conclusion, through comprehensive bioinformatics analysis, seven key SP-DEGs were identified in adipose tissue. DNMT1 was selected as the core gene during the development of metabolic syndrome. Therefore, moderately inhibiting DNMT1 expression in adipose tissue may be an effective treatment for metabolic syndrome.

## Supplementary Material

supplyment table secretory proteins.xlsx

supplyment table brownhubgenes.xlsx

## Data Availability

The Data generated during the study is available at 4TU.RearchData at : https://data.4tu.nl/private_datasets/gc8y4LfBIhsASzNQ-KSqFpVEvkVAfW8dbrFV8iUmP2c
